# A Voltammetric Electronic Tongue for the Resolution of Ternary Nitrophenol Mixtures

**DOI:** 10.3390/s18010216

**Published:** 2018-01-13

**Authors:** Andreu González-Calabuig, Xavier Cetó, Manel del Valle

**Affiliations:** 1 Sensors and Biosensors Group, Department of Chemistry, Universitat Autònoma de Barcelona, Edifici Cn, 08193 Bellaterra, Barcelona, Spain; andreu.gonzalez@uab.cat; 2 Future Industries Institute, University of South Australia, SA 5095 Adelaide, Australia; Xavier.CetoAlseda@unisa.edu.au

**Keywords:** electronic tongue, artificial neural networks, persistent pollutants, nitrophenols

## Abstract

This work reports the applicability of a voltammetric sensor array able to quantify the content of 2,4-dinitrophenol, 4-nitrophenol, and picric acid in artificial samples using the electronic tongue (ET) principles. The ET is based on cyclic voltammetry signals, obtained from an array of metal disk electrodes and a graphite epoxy composite electrode, compressed using discrete wavelet transform with chemometric tools such as artificial neural networks (ANNs). ANNs were employed to build the quantitative prediction model. In this manner, a set of standards based on a full factorial design, ranging from 0 to 300 mg·L^−1^, was prepared to build the model; afterward, the model was validated with a completely independent set of standards. The model successfully predicted the concentration of the three considered phenols with a normalized root mean square error of 0.030 and 0.076 for the training and test subsets, respectively, and *r* ≥ 0.948.

## 1. Introduction

The analysis of pollutants in wastewater, marine environments, and freshwater aquifers is of great importance to assure that pollutant levels stay within acceptable values. Among the most dangerous pollutants are nitrophenol compounds; these compounds are toxic inhibitors and of a persistent nature. The origin of nitrophenol compounds in the environment is anthropogenic; they originate in the degradation of dyes, pharmaceuticals, and pesticides. Among them, 4-nitrophenol is an especially toxic degradation product of parathion (O,O-diethyl O-(4-nitrophenyl)) phosphorothioate, a pesticide of extreme toxicity considered as hazardous waste and a high-priority toxic pollutant by the US Environmental Protection Agency and the European Chemical Agency [1,2].

Nitrophenols have been found in industrial wastewaters and in freshwater reservoirs and are present in the majority of marine environments. Traditional wastewaters and water purification plants have difficulty eliminating these compounds, as they require specific treatments with long incubation periods due to their high stability and solubility in water [3]. Hence, the development of sensors able to quantify the nitrophenol content in freshwater, wastewater, or marine environments is important.

**Figure 1:**
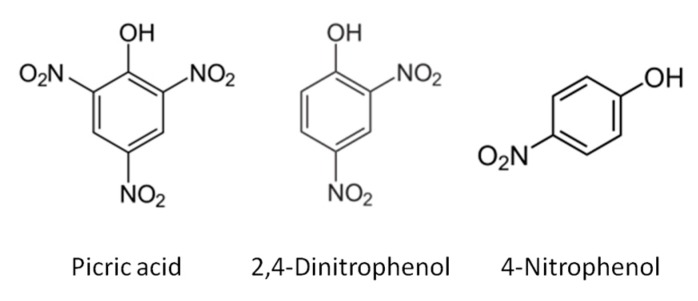
Chemical structures of picric acid, 2,4-dinitrophenol, and 4-nitrophenol.

Electrochemical sensors provide an excellent tool for performing on-site analysis at a reasonably cheap price with fast and robust results. Unfortunately, there are factors that hinder the applicability of such sensors in real samples, e.g., matrix effects, interferents, and electrode fouling. Recent reported works have approached the electrochemical detection of different nitrophenol compounds relying on the surface modification of electrodes subsequently used in voltammetry; poly(p-aminobenzene sulfonic acid) films [4], chitosan–ZnO nano-needles [5], and graphene oxide particles modified with chitosan and cyclodextrine [6], among others [7], have been used for this purpose. However, the reported works only focus on the detection of 4-nitrophenol or on the quantification of nitrophenol isomer mixtures. The work presented here will attempt to simultaneously quantify species having multiple and different nitro-groups such as picric acid, 2,4-dinitrophenol, and 4-nitrophenol (Figure 1).

In this context, the approach proposed is the use of an electronic tongue (ET) system [8]. ETs were initially postulated in the early 1990s to overcome limitations of single sensor methodologies and to make extensive use of sensor arrays and chemometric tools [9]. Since then, ETs have been used to resolve individual pollutants present in complex samples [10,11] and to correct interference and/or matrix effects in environmental determinations [12].

However, the use of sensor arrays, especially when sensors are voltammetric, generates highly complex data that cannot be treated by classical means; such data require previous pre-processing in order to extract meaningful information and allow proper modeling. The more common chemometric tools employed to process the chemical data are principal component analysis (PCA), partial least squares (PLS), and artificial neural networks (ANNs) [13]. The resulting approach, a sensor array plus chemometric tools, that forms the basis for ETs [14] is a simple way to tackle qualitative identification problems and quantitative determinations. Due to potential parallelisms with the human sense of taste [15], ETs have been extensively employed in the food and beverage field, with specific applications in wine analysis, such as the determination of its global characteristics, the prediction of the score given by an expert sensory panel, and the detection of adulterations [16,17,18,19]. Other application examples include wastewater monitoring, explosive detection, and discrimination and quantification of phenol isomers [20,21,22]. Using this methodology, it is possible to achieve the simultaneous quantification of different species in a mixture while diminishing effects of potential interfering agents through the use of chemometric data analysis techniques [23,24].

**Figure 2:**
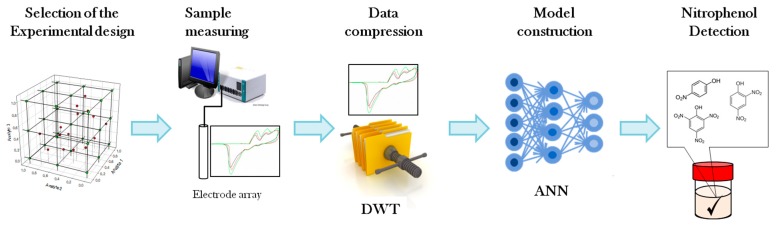
Scheme of the strategy followed in the resolution of nitrophenol-related compounds.

The proposed work performs a combined approach using electrochemical signals provided by a sensor array and chemometric data treatment in order to perform the simultaneous determination of three compounds with overlapped measurements. As shown in Figure 2, the use of cyclic voltammetry responses obtained from an array of epoxy graphite electrode plus metal electrodes is the sensor departure point. Discrete wavelet transform [25] was used to compress the data and to extract the chemically relevant information from original voltammograms, and ANNs were finally employed to build a response model to simultaneously predict the concentrations of picric acid, 4-nitrophenol, and 2,4-dinitrophenol content in aqueous solution mixtures, being the three compounds analytes of environmental concern.

## 2. Experimental

### 2.1. Reagents

The reagents used in this work were analytical reagent grade and all solutions were prepared using deionized water from a Milli-Q purification system (Millipore, Billerica, MA, USA). Potassium dihydrogenphosphate, potassium monohydrogenphosphate, picric acid, 2,4-dinitrophenol, and 4-nitrophenol were supplied by Sigma-Aldrich (St. Louis, MO, USA). KCl was supplied by Merck KGaA (Darmstadt, Germany).

### 2.2. Electronic Tongue

The voltammetric ET was formed by an array of 4 sensors, plus a combined reference Ag/AgCl and Pt counter electrode (Ingold, PT4805-S7/120). The ET approach departs from the signals from an array of electrodes; in this work, we intended to obtain an easy deployable sensor array for on-site situations. Thus, a quatrielectrode with external counter and reference electrodes was proposed. One-millimeter-diameter discs of platinum, silver, gold, and epoxy-graphite were used as working electrodes. The metal electrodes were fabricated from its metal wires, and the epoxy-graphite electrode was prepared from the known epoxy-graphite composite electrode [26] by mixing epoxy resin and carbon; afterwards, the electrodes were encased in inert epoxy resin (Epotek H77, Epoxy Technologies) using a PVC tube with a 6 mm inner diameter as the array body [27].

Electrochemical measurements were performed at room temperature (25 °C), using a 6-channel AUTOLAB PGSTAT20 (Ecochemie, The Netherlands) controlled through its GPES Multichannel 4.7 software package. A complete cyclic voltammogram was recorded for each sample and for each electrode by cycling the potential between −1.0 V and +1.0 V vs. Ag/AgCl with a step potential of 9 mV and a scan rate of 100 mV·s^−1^.

**Figure 3:**
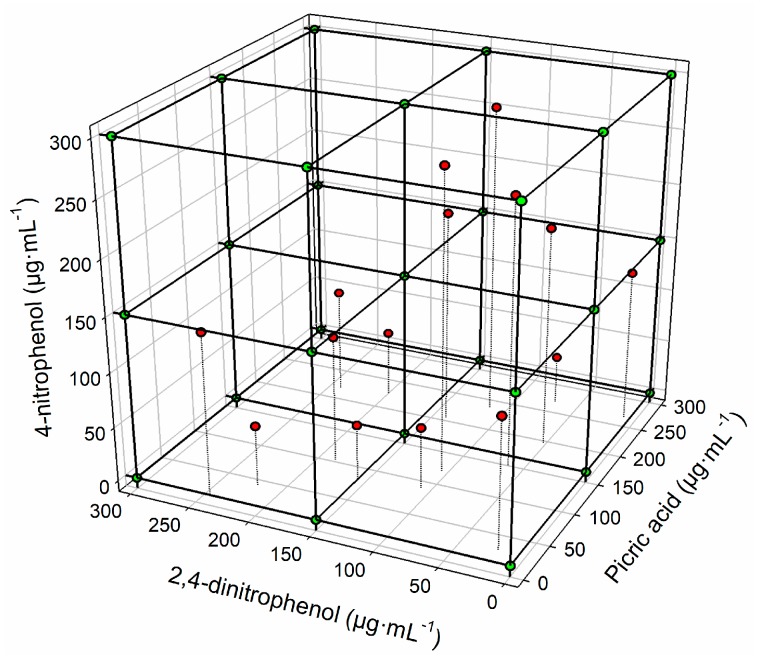
Representation of the full factorial experimental design employed, training samples (●, red circles), and test samples (●, green circles).

In order to get stable voltammetric responses and ensure reproducible signals from the array during the study, the electrodes were cycled in a phosphate buffer solution on the beginning of each sample measurement until a stable response was obtained; an electrochemical cleaning step was performed between samples at +1.2 V for 40 s in a cell containing 20 mL of 50 mM saline solution (0.1 M KCl) at pH 10 [28]. Figure 3 schematizes the distribution of the training and test samples in the three analytes considered.

### 2.3. Sample Preparation

The first step in the construction of the artificial neural network is the response model definition of the training and test subsets. In this case, the chosen experimental design for the train subset was a complete 3^3^ factorial design (27 samples) [29]; meanwhile, the validation of the constructed model was done with an external test set (12 samples), these were distributed randomly within the experimental domain (0 to 300 mg·mL^−1^ for each nitrophenol).

Samples were prepared in buffer (50 mM phosphate buffer solution at pH 6.5 and 50 mM KCl). Fresh stock solutions of nitrophenols were prepared the same day of the measurements.

### 2.4. Data Processing

The main objective of the first processing step is to simplify the input signal (4 sensors × 1784 current values at different potentials) without losing relevant chemical information; this step reduces training time, avoids redundancy in the input data, and allows for a model with better generalization ability [13].

**Figure 4:**
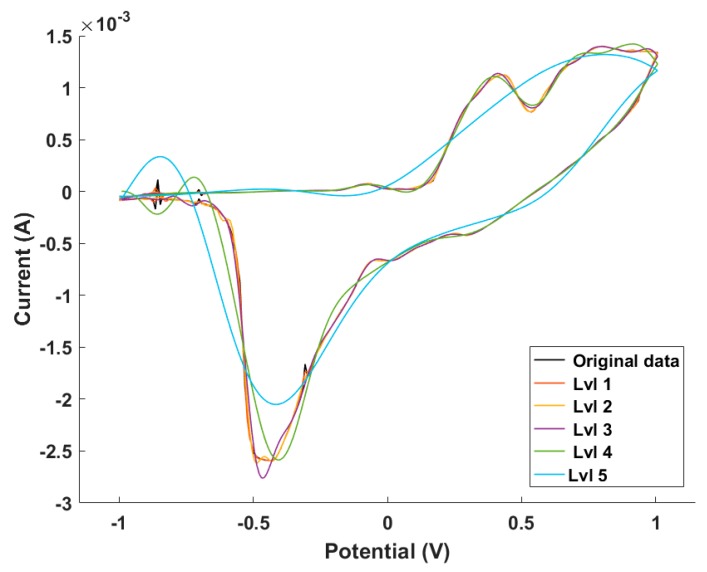
Reconstruction of the first 5 compression levels versus the original signal for cyclic voltammogram measured with the Ag electrode.

The compression of the voltammograms was achieved by means of discrete wavelet transform [25]: each voltammetric vector was compressed using Daubechies 3 mother wavelet and a 4th decomposition level. In this manner, the 1784 currents per sample were compressed to 132 coefficients per sample, achieving a 94.2% compression ratio. Figure 4 shows the reconstructed voltammetric signals for different compression levels.

The statistical treatment and data analysis was performed using routines written by the authors using MATLAB 2016b (MathWorks, Natick, MA, USA) employing its Neural Network and Statistical Toolboxes add-ons; Sigmaplot (Systat Software Inc., San Jose, CA, USA) was used to graphically represent and analyze the results.

## 3. Results and Discussion

### 3.1. Voltammetric Array Response

Voltammograms for each of the electrodes towards individual compounds were firstly evaluated to ensure that the generated signals are different enough and the obtained data is sufficiently rich to be the starting point for a multivariate calibration model.

**Figure 5:**
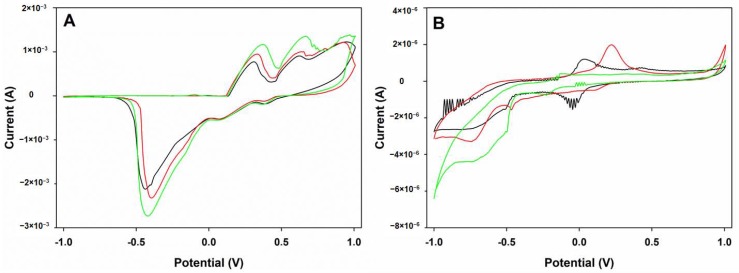

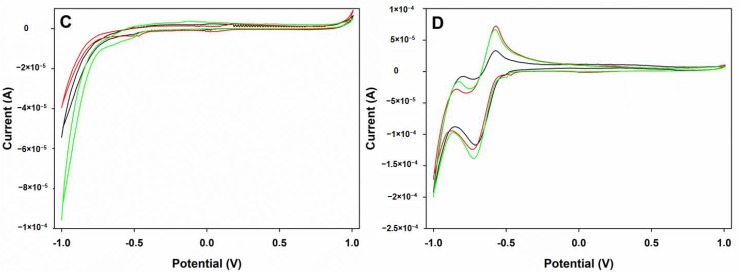
Cyclic voltammograms of picric acid (green), 2,4-dinitrophenol (black), and 4-nitrophenol (red), 50 μg·mL^−1^ individual solutions with (**A**) Ag electrode; (**B**) epoxy graphite electrode; (**C**) gold electrode; and (**D**) platinum electrode.

Therefore, following the conditions described in Section 2.2, individual stock solutions of 50 μg·mL^−1^ ppm of picric acid, 4-nitrophenol, and 2,4-dinitrophenol were analyzed (Figure 5). As a general trend, and as already reported in the literature [30,31], two processes were observed: the reduction of each nitrophenol group to its hydroxoamino form and the reversible redox oxidation of the hydroxoamino group to the nitrosophenol. In addition, slightly different signals were obtained for the different nitrophenol compounds, a necessary condition for an ET study.

Once it was confirmed that the different electrodes presented a differentiated electrochemical behavior towards the different nitrophenols under study, allowing for the differentiation of the three nitrophenols considered, the next step was the design of the proper ANN model architecture.

### 3.2. ANN Model Design

Once the data from the different subsets was collected, the voltammograms were compressed by use of DWT (compression details already determined), and the reduced dataset was fed to the different ANN models. For this, the next step was to choose the adequate ANN architecture; the habitual protocol to decide the details of an ANN configuration is trial-and-error given the complexity of an ANN and the number of parameters involved (learning strategy, learning parameters, number of layers, number of neurons in the hidden layer, transfer functions used, etc.) [32].

As already mentioned, the training subset samples were used to build the ANN models, and the performance of those models was estimated from the prediction of the analyte concentrations in the test subset samples. As the test subset is an external set that does not intervene in the modeling process, the goodness of fitting values for this subset is an unbiased parameter to evaluate the modeling performance.

After the compression of the obtained responses, the corresponding ANN configuration details were defined. First, it is needed to define which topology is necessary: the number of neurons in the input layer was equal to the number of DWT coefficients and the number of output neurons is determined by the number of compounds to be quantified. Hence, once the numbers of input and output neurons are defined, the number of neurons in the hidden layer and the type of transfer functions that will operate in the hidden and output layers need to be optimized. Normally, the use of a single hidden layer is sufficient to model common situations in analytical chemistry. All of these configuration parameters are optimized based on acquired experience working with ANNs and on a trial and error procedure, where the configuration that leads to the best performance is the one that is chosen [33].

The performance for each generated model was then evaluated with the external test subset by using the predictive abilities of the built model to predict concentrations of the nitrophenols present in the samples.

The main parameter used to evaluate the performance of the different models was the NRMSE (normalized root mean square error). The NRMSE is calculated for each of the configurations (a combination of hidden and output transfer functions and the number of neurons in the hidden layer) according to Equation (1), where X_expected_ is the theoretical concentration of the sample, X_predicted_ is the predicted concentration, j is the number of analytes considered, N the number of samples, and c_max_ is the maximum concentration.

(1) $$\mathrm{NRMSE}=\frac{\sqrt{\frac{\sum_{i}{\left(x_{\mathrm{expected}}-x_{\mathrm{predicted}}\right)}^{2}}{j\cdot N-1}}}{c_{\max}}$$

**Figure 6:**
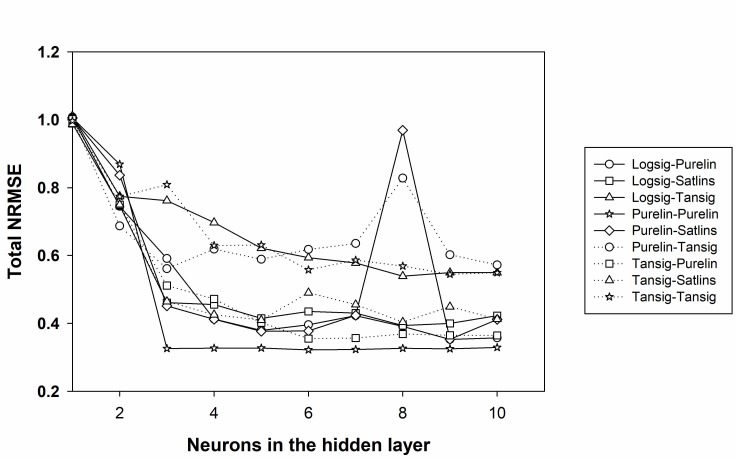
Normalized root mean square error (NRMSE) obtained for each transfer function combination and neurons in the hidden layer.

Thus, the best topology will be the one that gives the lowest NRMSE value. As can be observed in Figure 6, by plotting the total NRMSE versus the number of neurons, for all combinations assayed, this allows us to easily compare the performance of the models. Moreover, it clearly shows that purelin–purelin combination is the one with the lowest NRMSE. The plot also allows us to select the best number of neurons to use in the hidden layer, in this case architectures between 3 and 10 neurons in its hidden layer, seem to procure equivalent results.

However, the total NRMSE is not the only parameter evaluated. Comparison graphs of obtained vs. expected concentrations for the three quantified nitrophenols were built in order to visualize the predictive ability of the ANN model, separately for the training and the testing subsets, and its best linear fitting line was calculated. Afterwards, the best configuration could be selected taking into account the best results for NRMSE as well as the slope, intercept, and correlation coefficient values (considering that these should provide values as close as possible to ideal values of 1.0, 0.0, and 1.0 in that order).

After the evaluation of different topologies tested (90 different configurations in total), the finally selected ANN architecture was formed by an input layer of 136 neurons (4 sensors × 33 DWT coeffs.), a hidden layer with 7 neurons and purelin transfer function, and an output layer with 3 neurons and purelin transfer function. With this configuration it is possible to simultaneously determine the concentration of the ternary mixture of nitrophenols, picric acid, 2,4-nitrophenol, and 2-nitrophenol.

**Figure 7:**
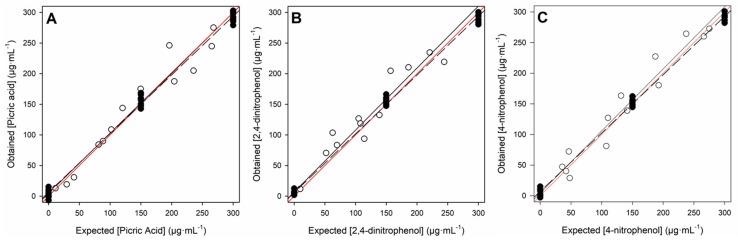
Obtained vs. expected concentrations plots for the training set (black points and dashed line) and the testing set (white points and solid line) for (**A**) picric acid; (**B**) 2,4-dinitrophenol; and (**C**) 4-nitrophenol.

As mentioned, comparison graphs of obtained vs. expected concentrations for the training and testing subsets, separately for each nitrophenol considered, were built to evaluate the predictive ability of the ANN model (see Figure 7). Both subsets provided a satisfactory linear trend, with fitted comparison lines having its parameters very close to the theoretical ones: slope and intercept equal to 1.0 and 0.0, respectively. As normally observed in practice, the training subset had the lowest NRMSE and better correlation coefficients (*r* ≥ 0.998), clearly improving the test subset (*r* ≥ 0.948); this was an expected difference as the training subset was the one employed to construct and select different model configurations, so the model was tailored to fit the training data, and the test subset was only used to evaluate the performance in the predictive capabilities of the model as an external subset of samples. The detailed results, described in Table 1, show satisfactory results for the test subset as the global NRSME for the three nitrophenols, which is as low as 0.076. 

To verify the correctness of the approach, a detailed numeric comparison of obtained vs. expected concentrations was performed, as summarized in Table 2. In there, the obtained values are listed together with those deduced from a classical single current measuring approach, a situation that will produce large errors if overlapping voltammetric signals occur, as it happens in this case. As can be seen in Table 2, the performance of the ET system clearly outweighs the single sensor approach, yielding values much closer to the theoretical ones, especially in the resolution of highly overlapped signals such as picric acid.

## 4. Conclusions

The presented work has shown the combination of a voltammetric array of four sensors with advanced chemometric processing, wavelet transform, and artificial neural networks to simultaneously quantify the concentrations of picric acid, 4-nitrophenol, and 2,4-dinitrophenol in aqueous solutions allowing for the resolution of complex mixtures with high overlapping peaks from different compounds. This study case clearly illustrates one of the capabilities of ET systems—the possibility of determining certain analyte counterbalancing any interfering species—but their response needed to be modeled.

The ET strategy allowed for the resolution of overlapping peaks and therefore the quantification of individual concentrations of nitrophenols; definitively, this consists in a very simple methodology that translates the complexity from the reactivity component or sensor component to the data treatment area, a field with increasing possibilities. This fact combined with the advantages of electrochemical sensors for on-field analysis results in a promising tool that could substitute the classical time-consuming methods, i.e., HPLC methodologies, and hopefully provide wastewater and water purification plants with a quick monitoring tool for these hazardous chemicals.

## Figures and Tables

**Table 1:** Results of the fitted regression curves for obtained vs. expected values, for the training and testing subsets of samples and the three considered nitrophenols (intervals calculated at the 95% confidence level).

Set	Analyte	r	Slope	Intercept (mg·L^−1^)	NRMSE	Total NRMSE
Training Set	picric acid	0.998	0.959 ± 0.027	6.3 ± 5.2	0.032	0.030
	2,4-dinitrophenol	0.998	0.961 ± 0.027	5.8 ± 10.4	0.032	
	4-nitrophenol	0.998	0.952 ± 0.021	7.1 ± 4.0	0.029	
Testing Set	picric acid	0.983	0.983 ± 0.161	4.6 ± 26.0	0.073	0.076
	2,4-dinitrophenol	0.948	1.012 ± 0.222	6.7 ± 30.2	0.087	
	4-nitrophenol	0.973	1.013 ± 0.160	4.4 ± 26.0	0.073	

NRMSE: normalized root mean square error.

**Table 2:** Compound concentrations found using the graphite epoxy composite electrode (single sensor approach) and the ET model for 5 water samples.

Sample	Expected Concentration (μg·mL^−1^)			Single Sensor Concentration (μg·mL^−1^)			ET Concentration (μg·mL^−1^)		
	picric acid	2,4-dinitro-phenol	4-nitro-phenol	picric acid	2,4-dinitro-phenol	4-nitro-phenol	picric acid	2,4-dinitro-phenol	4-nitro-phenol
1	102	186	107	275	151	110	109	210	81
2	11.1	244	141	392	222	26	12.9	219	139
3	265	9.9	131	208	67	8.1	245	11.7	163
4	204	138	187	362	196	73	187	132	227
5	81	157	42	270	148	49	84	205	40
